# Monthly Summary

**Published:** 1881-06

**Authors:** 


### MONTHLY SUMMARY.

*Care of the Teeth in Sickness.*—BY DR. FREDRICHS.—In an individual suffering from any of the acute febrile affections, in the first place, we have, as we all know, a complete suppression of the salivary secretions and a deposit upon the surface of the teeth and tongue, hardened masses of mucous. Now, should its removal be neglected, or as is the general rule, that absence, when we remove these masses, the teeth are covered with carious spots or furrows, and in some instances the caries has progressed to such an extent as to place the teeth beyond the vale of redemption—the result of neglect or ignoronce, in either case, equally culpable is the individual upon whom the responsibility of their preservation devolved.

The dentist is never consulted in such cases, until the patient has suffered days, and in some instances, weeks, of intense agony. Then comes, anæmic and emaciated, relating how much he has suffered and how many sleepless nights he has passed, to have some aching teeth extracted. The same individual will tell you that prior to this attack he never knew what it was to have an aching tooth.

Among children, the case is more serious. To see these little ones suffer, and especially where extraction has to be resorted to, knowing that the very remedy will be most apt to entail upon them future misery, is sad indeed. In these cases, the care of the teeth is entrusted to the attending physician alone and he should exert his best efforts to preserve them. No consideration whatever should so absorb his attention as to neglect to give them their proper share. From the beginning of an attack of this kind, the mucous accumulation should be daily removed from the teeth, which can be readily accomplished by means of a tooth-brush or a stick covered with some prepared chalk, followed by an alkaline gargle to be repeated several times a day. In all chronic diseases, dyspepsia, intestinal trouble, and particularly during pregnancy, the teeth deserve special attention. I would advise the strictest regard to cleanliness and a *constant* use of lime water.

Care should be observed in the administration of all acid medicines and in every instance they should be followed by an alkaline wash.—*Items of Interest.*

---

*Dr. Clowes on Plastic Fillings.*—Dr. J. W. Clowes, of New York, said in the recent meeting of the First District Dental Society, that he did not think it was necessary to say that, in his opinion, amalgam is a good filling, for he had said it so many times. He was, however, quite ready to admit that a poor amalgam makes a miserable filling. For example, amalgams that contain cadmium are a great curse to the profession and the public. Cadmium will act on a tooth very much as a rat acts on cheese. Scarce a day passes that he has not had come under his notice teeth filled with amalgam containing cadmium, and the evil caused by it is very wide-spread. Men who are high in the profession, and considered first-rate authorities, use amalgams containing cadmium, and with deplorable results. But though he had a great deal to say in praise of amalgams, yet as a filling it is far surpassed by gold. There is, however, a great difference between the two—any set of teeth can be saved with amalgam alone, but the same could not be claimed for gold alone. There are some cavities and positions in the mouth which cannot be reached for the insertion of gold fillings—that is the only thing against it. Amalgam is good and thoroughly reliable, and so is gold, and it is in their condemnation of these that the "new departure" men are wrong. He could not understand how men can use good amalgams, and have no confidence in them, unless it is that they do not know how to apply them properly. He hoped that their discussion would give them some light on the subject. They should strive to get good material. He used Lawrence's amalgam and thought it was the best. He had, however, tried recently an amalgam made by Dr. Welch, of Vineland, and was very much pleased with it. It seems to hold its color well; it hardens quickly, and goes in with a velvety touch. His experience of it was not long enough to decide definitely as to its merits, but so far, he considered it an improvement on anything he had tried.

The best phosphate he had used was Welch's, also. It works beautifully.—*Johnstons' Dental Miscellany.*

---

*Preliminary Prescribing.*—The *Philadelphia Medical Reporter*, in an article entitled "What to do when at a loss," collates a few useful ideas from a few medical writers. Dr. Weir Mitchell has remarked that a malady at its commencement only gives faint indications of its nature—the first letter or two of a word as it were—and the physician must wait patiently for other symptoms before he can hope to diagnose the case with confidence. But the patient expects him to do something meanwhile, and he must not seem to be in perplexity. The first rule is to *Enjoin rest.* This is in any case the most essential aid to treatment. But the patient will not be satisfied with that treatment alone. The doctor is expected to give something. Then comes in a good rule, the author of which is Dr. Robert Barnes— *When in doubt, give salines.* He says there is hardly any disease in which these will not do good in the beginning, nor hardly any in which they can possibly do harm. The next rule is to *Relieve any organ functionally disturbed.* This rule must be followed, however, with discretion. It is not always wise to purge when the bowels are constipated. An engorged liver is most promptly relieved by stimulating the functions of the kidneys, and so on. Fourthly, *Avoid committing yourself.*

This is obviously a great part of the tact of a skilful physician. He must not express a decided opinion before he is quite convinced, nor must he let it be seen that he has none. Lastly, he must always bear in mind the maxim of Hippocrates, *Do no harm.* This should guide, and, indeed, precede, every other idea. Mr. Francis Gallon, in his "Art of Travel," quotes a satirical remark, to the effect that there is a great difference between a good physician and a bad one, but very much less between a good physician and none at all. This is only another way of putting the advice last given.

---

*Chromograph*—Take 1,200 grains pure glycerine, 500 grains of gelatin, and enough barium sulphate to make it white. Pour a little boiling water on the gelatine in a jug, put the jug in boiling water and heat till the gelatine is entirely dissolved. When the solution is quite smooth pour it into a large evaporating basin, add the glycerine by degrees, boil well with constant stirring. Pour in barium sulphate mixed with water to a thin milk, and when white enough pour it into a thin dish the size of a large sheet of note-paper. The cover of a tin jujube box answers well. All air bubbles should be removed with a glass rod, but careful pouring will prevent their formation. Ink for the chromograph may be made by adding sufficient Judson's violet to any good ordinary ink. The writing to be copied should be even and distinct. Place the sheet of paper writing downwards on the composition, gently but firmly smooth it over the moist gelatinised surface by hand pressure, and rub so as to bring it well in contact. Remove the paper slowly from the top downwards, observing whether any portion may have missed the action of the ink, in which case gently rub for a second time the faulty space upon the composition. The fresh sheets on which copies are to be taken should be gently rubbed in a circular direction.

---

*To Restore India Rubber Goods.*—Dr. Pol (a Russian) recommends the following mixture as a bath for rubber goods which have lost their elasticity :—Water of ammonia, 1 part ; water, 2 parts ; in which the article should be immersed for a length of time, varying from a few minutes to one-half or one hour, until they resume their former elasticity, smoothness, and softness.

*Menthol.*—This new antiseptic and anti-neuralgic is stearoptene of peppermint, or menthol, a crystaline solid derived from the oil of the mentha piperita. It is not soluble in water, but dissolves readily in alcohol, ether or glycerine. A one to twenty solution may be obtained by adding one grain of menthol to six minims of alcohol with fourteen minims of water. Its antiseptic action resembles that of thymol; in the strength of 1 to 500 it will prevent the development of bacteria and kill those already in existence. Its anti-neuralgic action is obtained by painting it in solution (one grain of menthol in ten minims of alcohol) over the painful point. The author, McDonald, considers that menthol is the active antiseptic and anti-neuralgic principle of oil of peppermint.—*New York Med. Jour.*

---

*Relation of Tonsils to Virility.*—"I never exercise a male child's tonsils without explaining the possible effects to the parents, for I saw some years ago a statement made by a writer in one of the foreign quarterlies that amputation of the tonsils for the cure of chronic tonsillitis, was sure to destroy virility in a man. Some time after reading the article in question a medical friend happened to drop in, and, not at all satisfied in my own mind regarding the accuracy of the writer's statements, I put the question directly to him: "Do you believe that amputation of the tonsils destroys a man's virility?" "Oh no, Doctor," said he, "that is all trash; why I had my tonsils cut when I was a child." Gentlemen, my medical friend had been married twenty years; although his wife is an unusually healthy-looking woman, has never had a baby."—*Dr. Penson.*

---

*Charging for the "Know-how."*—A colored servant of a medical man in the South (*Pacific Med. and Surg. Journal*) to whom a patient, who had an important surgical operation performed, complained that his master had made a very steep charge of $25 for half an hour's work, when $5 would have been sufficient, replied, "He only charge you five dollars for de operation, de oder twenty was for de know-how."—*Louisville Med. News.*

